# Adverse event profile and associated factors following surgical voluntary medical male circumcision in two regions of Namibia, 2015–2018

**DOI:** 10.1371/journal.pone.0258611

**Published:** 2021-10-20

**Authors:** Gillian O’Bryan, Caryl Feldacker, Alison Ensminger, Magdaleena Nghatanga, Laura Brandt, Mark Shepard, Idel Billah, Mekondjo Aupokolo, Assegid Tassew Mengistu, Norbert Forster, Brigitte Zemburuka, Edwin Sithole, Gram Mutandi, Scott Barnhart, Gabrielle O’Malley

**Affiliations:** 1 Department of Global Health, International Training and Education Center for Health (I-TECH), University of Washington, Seattle, WA, United States of America; 2 Department of Global Health, University of Washington, Seattle, WA, United States of America; 3 Department of Global Health, International Training and Education Center for Health (I-TECH), University of Washington, Windhoek, Namibia; 4 Directorate of Special Programs-Ministry of Health and Social Services, Windhoek, Namibia; 5 Centers for Disease Control and Prevention (CDC/DDPHSIS/CGH/DGHT), Windhoek, Namibia; 6 Department of Medicine, University of Washington, Seattle, WA, United States of America; Centre for Sexual Health & HIV/AIDS Research (CeSHHAR)R, ZIMBABWE

## Abstract

**Introduction:**

Monitoring clinical safety of voluntary medical male circumcision (VMMC) is critical to minimize risk as VMMC programs for HIV prevention are scaled. This cross-sectional analysis describes the adverse event (AE) profile of a large-scale, routine VMMC program and identifies factors associated with the development, severity, and timing of AEs to provide recommendations for program quality improvement.

**Materials and methods:**

From 2015–2018 there were 28,990 circumcisions performed in International Training and Education Center for Health (I-TECH) supported regions of Namibia in collaboration with the Ministry of Health and Social Services. Two routine follow-up visits after VMMC were scheduled to identify clients with AEs. Summary statistics were used to describe characteristics of all VMMC clients and the subset who experienced an AE. We used chi-square tests to evaluate associations between AE timing, patient age, and other patient and AE characteristics. We used a logistic regression model to explore associations between patient characteristics and AE severity.

**Results:**

Of the 498 clients with AEs (AE rate of 1.7%), 40 (8%) occurred ≤2 days, 262 (53%) occurred 3–7 days, 161 (32%) between day 8 and 14, and 35 (7%) were ≥15 days post-VMMC. Early AEs (on or before day 2) tended to be severe and categorized as bleeding, while infections were the most common AEs occurring later (p<0.001). Younger clients (aged 10–14 years) experienced more infections, whereas older clients experienced more bleeding (p<0.001).

**Conclusions:**

Almost 40% of AEs occurred after the second follow-up visit, of which 179 (91%) were infections. Improvements in pre-surgical and post-surgical counselling and post-operative educational materials encouraging clients to seek care at any time, adoption of alternative follow-up methods, and the addition of a third follow-up visit may improve outcomes for patients. Enhancing post-surgical counselling and emphasizing wound care for younger VMMC clients and their caregivers could help mitigate elevated risk of infection.

## Introduction

Since 2008, voluntary medical male circumcision (VMMC) has been rapidly scaled-up as a key HIV prevention strategy after three randomized controlled trials found it decreases the risk of heterosexual HIV transmission to men by up to 60% [1–3]. The World Health Organization (WHO) and the Joint United Nations Programme on HIV/AIDS (UNAIDS) VMMC 2021 strategic framework set a goal of circumcising 90% of clients aged 10–29 years in 14 priority countries in Africa, targeting 5 million VMMCs annually [4]. In accordance with these international recommendations, in 2008 the Namibian Ministry of Health and Social Services (MoHSS) began scale-up of VMMC in Namibia [5]. In 2017, the MoHSS set a target to perform 300,000 VMMCs by 2022 [6].

Careful monitoring of VMMC clinical and programmatic quality is essential, particularly during rapid scale-up. Monitoring the proportion of VMMC clients who experience moderate or severe adverse events (AEs), as defined by WHO, is a commonly used indicator of clinical service delivery quality [7]. Combined moderate and severe AE rates above 2% signal a need for programmatic review and potential corrective action [7]. AE rates reported during VMMC randomized controlled trials and from active surveillance settings range from 0.5%–8.0%, with limited and inconsistent results reported from routine program implementation [1–3, 8–21].

Scheduled routine follow-up visits in VMMC programs help ensure patient safety by identifying, treating, and managing surgical complications. The initial 2009 WHO guidance did not specify a follow-up schedule, so during initial implementation the MoHSS instituted the two-visit follow-up schedule [22]. The WHO later released updated guidance recommending a third follow-up visit; however, Namibia did not implement a third follow-up visit to mitigate stress on the primary healthcare system and because of successful and safe program implementation [23]. The MoHSS policy recommends follow-up visits on day 2 (visit 1) and day 7 (visit 2) after VMMC to verify healing and to identify AEs for immediate attention [5]. Data from other sub-Saharan African countries, however, indicate that adherence to scheduled visits is suboptimal, and AEs may occur after the last scheduled visit [8, 15, 19, 24, 25].

The International Training and Education Center for Health (I-TECH) supported VMMC scale-up in two regions of Namibia with high HIV prevalence and low VMMC coverage among boys and men aged 15–29 years. I-TECH began providing VMMC technical assistance to the MoHSS in October 2014 and expanded to direct service delivery of VMMC in 2015. At group and individual counselling sessions on the day of operation, VMMC clients were instructed to return to the clinic for the day 2 and day 7 follow-up visits, and at any time if they experienced an AE. I-TECH also implemented MoHSS guidance to trace clients who did not return for visits 1 and 2.

The objective of this study was to describe the AE profile from routine program implementation of the surgical VMMC program in the I-TECH supported regions Oshana and Zambezi (January 2015–August 2018). This study includes only data from surgical VMMC as device-based circumcision was not piloted in Namibia until 2019 and has not yet been approved by national authorities. Using aggregate program data on VMMC implementation, complemented by an aggregate database including only individuals with AEs, we investigated the AE profile of the VMMC program and identified individual factors associated with the development, severity, or timing of AEs. Identifying AE profiles and risk factors in Namibia could help improve program quality within the studied regions and nationally.

## Materials and methods

### Ethics

This study obtained required approvals and was conducted under a routine data use protocol jointly approved by the Centers for Disease Control and Prevention (CDC) Office of the Associate Director for Science (OADS) and the Office of the Executive Director of the Namibian MoHSS. The information included in this cross-sectional analysis was routinely collected, program data. The study received a non-research determination from the University of Washington institutional review board (IRB) and was reviewed in accordance with CDC human research protection procedures and was determined to be a non-research, public health program activity by CDC OADS. In Namibia, adults <18 years must obtain written approval from a parent or guardian for surgical procedures. No additional verbal or written consent from patients was sought for this secondary analysis of de-identified data.

### Data collection

For this analysis, we created a dataset without individual identifiers from line lists of individual moderate and severe AEs kept by clinicians at the two main regional sites. The line listing included details on each moderate and severe AE, including client age, AE timing, AE severity, AE type, AE management, and AE outcomes. Surgical type (dorsal slit or forceps guided) was not included.

MoHSS monthly program summary reports compiled from site-level VMMC registers and client forms by I-TECH’s site-level strategic information staff were used to establish denominator of number of VMMCs performed. The summary report data were disaggregated by location, PEPFAR age groups, and surgical type.

### Definitions

Two surgical circumcision techniques were used over the study period. Forceps-guided VMMC was approved for clients ages 15 and above; for clients ages 10 and above, only dorsal slit VMMC was approved. Clinicians determined the surgical type for clients aged 15 and above. In Namibia, VMMC for clients aged 10–14 and the dorsal slit surgical procedure were authorized by the MoHSS in October 2016, and the use of the forceps guided procedure was discontinued in Namibia in July 2019. Generally, severe AEs require surgical intervention or hospitalization. Any AE not classified as severe but that required medical intervention was considered moderate [7, 26]. Mild AEs were not reported nor included in this analysis. AEs were categorized according to standard WHO definitions of bleeding, infection and wound disruption, and other AEs (e.g., excessive swelling, hematoma) [7]. Among the few clients who experienced more than one category of AE, only the most severe AE was included in this analysis. Follow-up visits 1 and 2 conformed to Namibia MoHSS guidelines [22]. We used PEPFAR age categories for consistency and to facilitate comparison with another PEPFAR program analysis from Zimbabwe: 10–14 years; 15–19 years; and ≥20 years [27, 28]. Timing of AE was determined using the number of days between surgery (day 0) and the day the AE was diagnosed at the clinic. Timing of AEs was categorized for this study as ≤2 days, 3–7 days, 8–14 days, and ≥15 days post-surgery [28]. Care seeking delay was determined using the number of days between the day the client reported noticing the AE and the day the AE was diagnosed at the clinic.

### Data analysis

We used summary statistics to describe the demographics of patients by region compiled from the routine program data of the 28,990 clients who underwent VMMC during the implementation period as well as the 498 clients who experienced an AE (as identified by the line lists). Chi-square tests were used to evaluate associations between the timing of AEs, client age, and both patient and AE characteristics. A logistic regression model was used to explore associations between patient characteristics and AE severity. Analyses were conducted using STATA. P-values <0.05 were considered statistically significant.

## Results

### Demographics and AE characteristics

During the study period, 28,990 VMMCs were performed in two regions (Oshana and Zambezi; Table 1) with 498 clients documented in the line lists as experiencing an AE (Table 2).

**Table 1:** Characteristics of all Voluntary Medical Male Circumcision (VMMC) clients (N = 28,990) in the Oshana and Zambezi regions in Namibia (January 2015–August 2018).

Characteristics	Circumcised Clients n (%)
Year	
FY15 (Jan.-Sep. 2015)	6,251 (22%)
FY16 (Oct. 2015-Sep. 2016)	7,686 (27%)
FY17 (Oct. 2016-Sep. 2017)	8,004 (28%)
FY18 (Oct. 2017-Aug. 2018)	7,049 (24%)
Age, years	
10–14	8,543 (29%)
15–19	10,083 (35%)
20+	10,364 (36%)
Surgical type*	
Dorsal slit	9,210 (33%)
Forceps guided	19,094 (67%)
Region	
Zambezi	11,183 (39%)
Oshana	17,807 (61%)
Season, months	
Oct–Dec	2,806 (10%)
Jan–Mar	4,117 (14%)
Apr–Jun	13,254 (46%)
Jul–Sep	8,813 (30%)

*686 missing surgical type.

**Table 2:** Characteristics of Voluntary Medical Male Circumcision (VMMMC) clients with Adverse Events (AEs) by region in Namibia (January 2015-August 2018).

	Zambezi (n = 220)	Oshana (n = 278)	Total (n = 498)
	N (%)*	N (%)*	N (%)*
Age, years			
Mean	20.8	22.5	21.7
Median (IQR)	18 (15, 24)	21 (16, 26)	20 (16, 26)
Age group, years			
10–14	34 (15%)	31 (11%)	65 (13%)
15–19	94 (43%)	86 (31%)	180 (36%)
≥20	92 (42%)	161 (58%)	253 (51%)
AE severity			
Moderate	192 (87%)	192 (69%)	384 (77%)
Severe	28 (13%)	86 (31%)	114 (23%)
Facility site type			
Static	178 (81%)	181 (65%)	359 (72%)
Outreach	42 (19%)	97 (35%)	139 (28%)
Days from VMMC to AE diagnosis			
Mean	8.9	7.4	8.1
Median (IQR)	7 (7, 11)	7 (5, 9)	7 (6, 9)
≤2	7 (3%)	33 (12%)	40 (8%)
3–7	115 (52%)	147 (53%)	262 (53%)
8–14	80 (36%)	81 (29%)	161 (32%)
≥15	18 (8%)	17 (6%)	35 (7%)
Care seeking delays			
Mean	3.0	1.7	2.2
Median (IQR)	3 (2, 4)	1 (0, 2)	2 (1, 3)
Same day	14 (6%)	78 (28%)	92 (18%)
After 1–3 days	142 (65%)	168 (60%)	310 (62%)
After 4+ days	64 (29%)	32 (12%)	96 (19%)
PEPFAR AE type			
Bleeding	2 (1%)	37 (13%)	39 (8%)
Infection/wound disruption	192 (87%)	207 (74%)	399 (80%)
Other (swelling/hematoma)	26 (12%)	34 (12%)	60 (12%)
Season, months			
Oct–Dec	14 (6%)	23 (8%)	37 (7%)
Jan–Mar	42 (19%)	43 (15%)	85 (17%)
Apr–Jun	94 (43%)	135 (49%)	229 (46%)
Jul–Sep	70 (32%)	77 (28%)	147 (30%)

Abbreviations: IQR, Interquartile range (Q1, Q3); PEPFAR, U.S. President’s Emergency Plan for AIDS Relief.

*Percentages are within-category column proportions.

The majority of VMMCs were completed in the Oshana region (61%). VMMC rates varied seasonally, with 46% of VMMCs conducted between April and June, which corresponds to Namibia’s coolest months and some school holidays. According to traditional beliefs, conducting VMMC procedures during this season promotes faster healing.

Over the implementation period, 29% of VMMCs were performed among clients aged 10–14 years, 35% among clients aged 15–19 years, and 36% among clients aged ≥20 years. The MoHSS issued a circular in October 2016 expanding VMMC service provision to clients aged 10–14 years based on the 2013 demographic health survey finding that 13.4% of Namibians aged 15 years reported engaging in sexual intercourse [29]. Before October 2016, no VMMCs were performed for clients aged 10–14 years.

Among the 498 clients with a moderate or severe AE (AE rate of 1.7%) (Table 2), the mean age was 21.7 years and the median was 20 years. VMMCs were performed at both static hospital sites (72%) and outreach sites (28%) staffed by mobile outreach teams. The median time from surgery to AE diagnosis was 7 days, with more than 39% of AEs being diagnosed on or after day 8. Men who noted a potential AE waited an average of 2.2 days before seeking care, and 19% of men waited 4 or more days before seeking care. Infections were the most common type of AE (80%).

### Associations between client and AE characteristics and time to AE diagnosis

Several client and AE characteristics were associated with AE timing (Table 3). First, AE severity varied significantly by time to AE diagnosis (p<0.001); although over 52% of both moderate and severe AEs occurred on days 3 through 7, severe AEs were more common in the early post-operative period, with 23% of all severe AEs occurring on or before day 2 (visit 1) compared to only 4% of moderate AEs. This is likely due to bleeding events: 46% of bleeding AEs occurred on or before day 2 and 87% occurred within 7 days. Almost 25% and 44% of severe and moderate AEs, respectively, occurred on day 8 or later (after the second scheduled follow-up visit). AE type also differed over time to AE diagnosis (p<0.001). Of AEs occurring on or after day 8, the vast majority were infections. The timing of AEs did not differ by age group, region, or season.

**Table 3:** Chi square results of associations by time to Adverse Event (AE) diagnosis (N = 498) among men who underwent Voluntary Medical Male Circumcision (VMMC) in Namibia (January 2015–August 2018).

	≤ Day 2	Days 3–7	Days 8–14	Day 15+	p-value
	≤ Visit 1	≤ Visit 2	>Visit 2	>Visit 2	
	n (%)	n (%)	n (%)	n (%)	
Age group, years					0.11
10–14	3 (5%)	39 (60%)	18 (28%)	5 (8%)	
15–19	14 (8%)	107 (59%)	48 (27%)	11 (6%)	
≥20	23 (9%)	116 (46%)	95 (38%)	19 (8%)	
AE severity					<0.001
Moderate	14 (4%)	202 (53%)	137 (36%)	31 (8%)	
Severe	26 (23%)	60 (53%)	24 (21%)	4 (4%)	
Care seeking delays					<0.001
Same day	22 (24%)	50 (54%)	18 (20%)	2 (2%)	
After 1–3 days	16 (5%)	190 (61%)	95 (31%)	9 (3%)	
After ≥4 days	2 (2%)	22 (23%)	48 (50%)	24 (25%)	
PEPFAR AE type					<0.001
Bleeding	18 (46%)	16 (41%)	3 (8%)	2 (5%)	
Infection/wound disruption	7 (2%)	213 (53%)	147 (37%)	32 (8%)	
Other (swelling/hematoma)	15 (25%)	33 (55%)	11 (18%)	1 (2%)	
Region					0.003
Zambezi	7 (3%)	115 (52%)	80 (36%)	18 (8%)	
Oshana	33 (12%)	147 (53%)	81 (29%)	17 (6%)	
Season, month					0.35
Oct–Dec	5 (14%)	19 (51%)	12 (32%)	1 (3%)	
Jan–Mar	8 (9%)	41 (48%)	32 (38%)	4 (5%)	
Apr–Jun	13 (6%)	121 (53%)	79 (35%)	16 (7%)	
Jul–Sep	14 (10%)	81 (55%)	38 (26%)	14 (10%)	

Abbreviations: PEPFAR, U.S. President’s Emergency Plan for AIDS Relief.

### Associations between client age and AE characteristics

AE type differed by client age (p<0.001); infection was more common in boys aged 10–14 years (16%) and bleeding was more common in clients aged ≥20 years (74%; Table 4). Clients aged ≥15 years were more likely to have a severe AE than those aged 10–14 years (p = 0.04). Facility type and season varied significantly by client age (p<0.001). More AEs occurred among clients aged 15–19 years who underwent VMMC at outreach sites between October and December and also between April and June, which corresponds to high-volume outreach campaign efforts during school holidays. In the Zambezi region, more clients aged 10–14 years underwent VMMC, whereas more clients aged ≥20 years underwent VMMC in the Oshana region (p = 0.002). Client age was not significantly associated with time to AE diagnosis or delays in care seeking.

**Table 4:** Chi square results of associations between age group and Adverse Event (AE) characteristics among men (N = 498) who underwent Voluntary Medical Male Circumcision (VMMC) in Namibia (January 2015–August 2018).

	Age, years			
	10–14	15–19	≥20	p-value
	n (%)	n (%)	n (%)	
AE severity				0.04
Moderate	58 (15%)	137 (36%)	189 (49%)	
Severe	7 (6%)	43 (38%)	64 (56%)	
Facility site type				<0.001
Static	60 (17%)	108 (30%)	192 (53%)	
Outreach	5 (4%)	72 (52%)	62 (45%)	
Days from VMMC to AE diagnosis				0.11
0–2	3 (8%)	14 (35%)	23 (58%)	
3–7	39 (15%)	107 (41%)	116 (44%)	
8–14	18 (11%)	48 (30%)	95 (59%)	
≥15	5 (14%)	11 (31%)	19 (54%)	
Care seeking delay				0.44
Same day	8 (9%)	31 (34%)	53 (58%)	
After 1–3 days	46 (15%)	111 (36%)	153 (49%)	
After ≥4 days	11 (12%)	38 (40%)	47 (49%)	
PEPFAR AE type				0.001
Other	2 (3%)	28 (47%)	30 (50%)	
Bleeding	0 (0%)	10 (26%)	29 (74%)	
Infection	63 (16%)	142 (36%)	194 (49%)	
Region				0.002
Zambezi	34 (16%)	94 (43%)	92 (42%)	
Oshana	31 (11%)	86 (31%)	162 (58%)	
Season, months				<0.001
Oct–Dec	5 (14%)	20 (54%)	12 (32%)	
Jan–Mar	15 (18%)	20 (24%)	50 (59%)	
Apr–Jun	29 (13%)	100 (44%)	100 (44%)	
Jul–Sep	16 (11%)	40 (27%)	91 (62%)	

Abbreviations: PEPFAR, U.S. President’s Emergency Plan for AIDS Relief.

### Factors associated with AE severity

Using aggregate data, we explored associations between client and AE characteristics and the risk of experiencing a severe versus moderate AE (Table 5). January–March (odds ratio [OR]: 4.78) and July–September (OR: 4.01) had higher odds of severe AEs related to VMMC compared to October–December. After grouping by facility, we found that bleeding AEs were 16.19 times more likely to be severe than other PEPFAR AE types. Of the 39 AEs categorized as bleeding during the implementation period, 37 (95%) occurred in the Oshana region. The Oshana region had 2.31 times higher risk of severe AEs compared to Zambezi. We did not detect an association between AE severity and age or AE timing.

**Table 5:** Factors associated with experiencing a severe Adverse Event (AE) after Voluntary Medical Male Circumcision (VMMC) in Namibia (January 2015–August 2018)*.

	Univariate OR (95% CI)	p-value	Multivariable OR (95% CI)	p-value
Age Group, years				
10–14	1.00 (ref)		1.00 (ref)	
15–19	2.60 (0.60–11.32)	0.20	1.60 (0.44–5.91)	0.48
≥20	2.81 (0.96–8.23)	0.06	1.07 (0.33–3.42)	0.91
Days from VMMC to AE				
≤2	1.00 (ref)		1.00 (ref)	
3–7	0.16 (0.07–0.39)	<0.001	0.93 (0.34–2.53)	0.89
8–14	0.09 (0.04–0.24)	<0.001	0.70 (0.26–1.93)	0.50
≥15	0.07 (0.03–0.17)	<0.001	0.29 (0.08–1.09)	0.07
Care seeking delays				
Same day	2.49 (1.51–4.11)	<0.001	0.65 (0.24–1.75)	0.40
After 1–3 days	0.97 (0.50–1.88)	0.93	0.47 (0.24–0.95)	0.04
After ≥4 days	1.00 (ref)		1.00 (ref)	
PEPFAR AE type				
Other (swelling/hematoma)	1.00 (ref)		1.00 (ref)	
Bleeding	17.31 (5.04–59.44)	<0.001	16.19 (4.75–55.21)	<0.001
Infection/wound disruption	0.12 (0.09–0.17)	<0.001	0.14 (0.09–0.22)	<0.001
Region				
Zambezi	1.00 (ref)		1.00 (ref)	
Oshana	3.07 (1.81–5.21)	<0.001	2.31 (1.42–3.75)	0.001
Season, months				
Oct–Dec	1.00 (ref)		1.00 (ref)	
Jan–Mar	4.46 (2.00–9.96)	<0.001	4.78 (1.15–19.79)	0.03
Apr–Jun	3.17 (1.15–8.70)	0.03	3.46 (0.77–15.52)	0.12
Jul–Sep	3.81 (2.06–7.05)	<0.001	4.04 (1.29–12.68)	0.02

Abbreviations: OR, odds ratio; CI, confidence interval; PEPFAR, U.S. President’s Emergency Plan for AIDS Relief.

*Clustered by facility code.

## Discussion

Safe VMMC services are essential for HIV epidemic control. Increased understanding of the AE profile of large-scale VMMC programs can help improve service delivery and patient safety. In over 15 quarters of VMMC program implementation by I-TECH in Namibia, 1.7% of clients experienced a moderate or severe AE. This rate is higher than the reported AE rates of some other large-scale VMMC programs in sub-Saharan Africa, but is lower than rates observed in clinical trial settings and well within the commonly accepted 2% AE rate for program safety [1–3, 7, 25, 26]. The significant differences in patterns of AE severity, timing, and client age, similar to other VMMC research and implementation settings, warrant attention. Our findings can inform improvements in program implementation and can ensure client safety during VMMC scale-up.

Bleeding was the most frequent early surgical AE, whereas infection was the most common late onset AE. These results are comparable to those reported in other studies [19, 28, 30]. Bleeding AEs often require surgical exploration to correct and are therefore categorized as severe [7]. The high number of bleeding AEs in the Oshana region likely contributed to this region having a two times higher risk of severe AEs than the Zambezi region. More bleeding AEs in Oshana could be due to the higher volume of circumcisions performed, better reporting, or the slightly older population of VMMC clients in Oshana during the implementation period (41% of clients were aged ≥20 years in Oshana versus 29% in Zambezi).

Our findings are similar to those of a study on AE patterns in Zimbabwe’s VMMC program, which found infections were more frequent in clients aged 10–14 years while bleeding was more frequent in clients aged ≥20 years [28]. More frequent bleeding in older clients could be related to increased vascularity of the penile skin, or to early resumption of sex following VMMC, which increases the risk of AEs [31].

Although infection adverse events were more frequent in clients aged 10–14 years in our findings, they represent an AE rate of only 0.7%, well under the 2% acceptable global threshold for program quality and corrective action. This low AE rate should be considered in combination with the most recent program guidance from PEPFAR changing the lower age limit for VMMC to 15 years and WHO guidelines which provide key considerations regarding policy formulation on offering VMMC to younger adolescents 10–14 years including public health burden of HIV and impact on HIV incidence, human rights guidance and consenting procedures, safety, and preferences regarding traditional male circumcision [32, 33]. Additionally, the latest WHO recommendations include use of WHO-prequalified male circumcision devices as an alternative to surgical VMMC for 10–14 year olds in keeping with the decision of whether to offer VMMC to younger adolescents [32]. Infection in younger VMMC clients in Namibia and other settings may be attributed to poor hygiene, misunderstanding of post-operative instructions leading to improper wound care, lack of parent or guardian availability or support, or inability to identify and report abnormal healing [8, 28, 34–37]. Potential interventions that could further mitigate the risk of infection in younger age groups include age-appropriate post-surgical counselling, increased engagement of guardians, and provision of supplies to support proper hygiene (e.g., salt, cotton swabs) [28, 38–41].

Although most AEs occurred before follow-up visit 2 (day 7), 39% of AEs occurred after this scheduled visit. AE identification at 7 or more days after VMMC is not uncommon. An analysis of a similar large-scale VMMC program in Zimbabwe found that 23% of surgical AEs were diagnosed after follow-up visit 2, whereas a study in Kenya found clients experienced infections 9 days after VMMC and bleeding 6.7 days after VMMC [19]. A prospective study in South Africa found a mean time to AE diagnosis of 7 days [10]. Additional post-surgical counselling could address these late onset AEs and follow-up visit retention by reinforcing the importance of seeking care at any time and by educating clients on the warning signs of poor healing to improve client care seeking behaviors even after scheduled visits [28]. Alternative methods of follow-up, such as using mobile communication technology, could be considered as a proxy for active surveillance to confirm final healing, reduce provider workload, and improve follow-up visit retention [42]. Many programs implement a third scheduled follow-up visit at either day 21 or 42 post circumcision; however high attrition rates for VMMC follow-up have been found, particularly for the third follow-up visit [10, 19, 28].

In our study, most men with severe AEs rapidly sought care. Most clients with an AE (62%) waited only 1–3 days between noting potential complications and seeking care. Delays were shortest for men with severe AEs. This suggests clients knew the signs of complications and swiftly sought care and there was also broad service availability. Most VMMC procedures occurred at static sites during the implementation period, and transportation to the facility was provided, which improved accessibility and continuation of care. Differences in care-seeking delays by region could reflect differences in ease of service access. We found that the mean delay in seeking care was 1.7 days in the Oshana region compared to 3.0 days in the Zambezi region. Oshana is a small region with a centrally located regional hospital, and transportation is relatively easy; however, Zambezi has several transportation challenges, including greater distances, seasonal flooding, and at times, compromised road conditions, which potentially hinders healthcare access. The Zambezi region also has a higher proportion of people in the lowest wealth quintile compared to the Oshana region, so patient there may not be able to afford VMMC-associated non-clinical auxiliary (e.g., transportation) costs [29].

This study has several limitations. Analysis of the association between HIV status and AE timing and other AE and client characteristics was not possible for this study as HIV status was not included in the monthly AE line-listing. Second, surgical type was not included in the monthly AE line-listing; therefore, analysis of the association between surgical type and AE timing was not possible. Clinicians in the I-TECH supported VMMC program received certification in the dorsal slit surgical procedure on 15 December 2016 and subsequently began using only the dorsal slit procedure for clients 10–14 years. Promotion of the dorsal slit method for all VMMCs for added safety, regardless of client age, was implemented in July 2019 following MoHSS guidance and reinforced by PEPFAR guidance in 2020, after this study’s implementation period [43, 44]. Therefore, for clients older than 14, it was up to the discretion of the clinician which surgical type to use. Third, we could not determine whether follow-up visit attendance was related to identification and timely treatment of AEs as routine attendance of follow-up visits was not included in the monthly MoHSS AE line-listing. This form of passive reporting, relying on men to seek care for a concern and for a clinician to report the AE, is likely to result in underreporting of AEs in Namibia and elsewhere [23, 38]. Lastly, care-seeking delays were calculated using clients’ report of AE onset, which may have been subject to recall bias.

## Conclusions

Clinical safety of VMMC is critical as this intervention is scaled up to meet the ambitious WHO and UNAIDS targets of 90% coverage among boys and men aged 10–29 years. Our findings support several programmatic recommendations that may further improve clinical service quality in the Namibia VMMC program and other VMMC programs. Emphasizing signs of abnormal healing and advising clients to seek care at any time and promptly could help improve patient outcomes; in our study, many AEs occurred after the second follow-up visit (day 7). Ensuring that printed post-operative materials provided to all clients are accessible to literate and illiterate clients could also improve outcomes. Namibia could consider adding a third follow-up visit at day 21 or 42, although this has the potential to stress the healthcare system for marginal gains in client safety; improving follow-up counselling and client materials may be more effective strategies. Alternative follow-up methods using mobile communication technology could help confirm client healing, reduce provider workload, and improve follow-up visit adherence. Age-appropriate surgical counselling could help reduce the risk of infection and ensure safety of clients aged 10–14 years and could include a review of post-surgical expectations during the consent process with parents and caregivers. The Namibian MoHSS guidance released in 2016 requiring use of the dorsal slit procedure in VMMC clients aged less than 15 and discontinuing use of the forceps guided procedure for all VMMC clients in 2019 both contributed to improved clinical safety. Because of the high proportion of infection events in the youngest clients among the AEs observed in our study, Namibia could consider updating its guidelines to reflect PEPFAR guidance to increase the age of VMMC eligibility to 15 years and WHO guidance providing key considerations in the decision to offer surgical or device-based VMMC to younger adolescents 10–14 years [32, 33].

## Supporting information

S1 Dataset Study dataset. (TXT)
